# Next generation sequencing of high-grade adult-type diffuse glioma in the Netherlands: interlaboratory variation in the primary diagnostic and recurrent setting

**DOI:** 10.1007/s11060-024-04568-8

**Published:** 2024-01-29

**Authors:** Mark P. van Opijnen, Marike L. D. Broekman, Edwin Cuppen, Hendrikus J. Dubbink, Arja ter Elst, Ronald van Eijk, Angelika Mühlebner, Casper Jansen, Robert van der Geize, Ernst-Jan M. Speel, Patricia J. T. A. Groenen, Filip Y. F. de Vos, Pieter Wesseling, Wendy W. J. de Leng, Sybren L. N. Maas

**Affiliations:** 1 Department of Neurosurgery, Haaglanden Medical Center, The Hague, The Netherlands; 2 Department of Neurosurgery, Leiden University Medical Center, Leiden, The Netherlands; 3 Hartwig Medical Foundation, Amsterdam, The Netherlands; 4 Center for Molecular Medicine and Oncode Institute, University Medical Center Utrecht, Utrecht, The Netherlands; 5 Department of Pathology, Erasmus MC Cancer Institute, University Medical Center Rotterdam, Rotterdam, The Netherlands; 6 Department of Pathology and Medical Biology, University Medical Center Groningen, Groningen, The Netherlands; 7 Department of Pathology, Leiden University Medical Center, Leiden, The Netherlands; 8 Department of Pathology, University Medical Center Utrecht, Utrecht, The Netherlands; 9 Laboratorium Pathologie Oost-Nederland, Hengelo, The Netherlands; 10 Department of Pathology, GROW-School for Oncology and Reproduction, Maastricht University Medical Center, Maastricht, The Netherlands; 11 Department of Pathology, Radboud University Medical Center, Nijmegen, The Netherlands; 12 Department of Medical Oncology, Utrecht University Medical Center, Utrecht, The Netherlands; 13 Department of Pathology, Amsterdam University Medical Centers, Amsterdam, The Netherlands; 14 Princess Máxima Center for Pediatric Oncology, Utrecht, The Netherlands

**Keywords:** Next generation sequencing, High-grade glioma, Adult, Variations, Practice

## Abstract

**Purpose:**

Next generation sequencing (NGS) is an important tool used in clinical practice to obtain the required molecular information for accurate diagnostics of high-grade adult-type diffuse glioma (HGG). Since individual centers use either in-house produced or standardized panels, interlaboratory variation could play a role in the practice of HGG diagnosis and treatment. This study aimed to investigate the current practice in NGS application for both primary and recurrent HGG.

**Methods:**

This nationwide Dutch survey used the expertise of (neuro)pathologists and clinical scientists in molecular pathology (CSMPs) by sending online questionnaires on clinical and technical aspects. Primary outcome was an overview of panel composition in the different centers for diagnostic practice of HGG. Secondary outcomes included practice for recurrent HGG and future perspectives.

**Results:**

Out of twelve neuro-oncology centers, the survey was filled out by eleven (neuro)pathologists and seven CSMPs. The composition of the diagnostic NGS panels differed in each center with numbers of genes ranging from 12 to 523. Differences are more pronounced when tests are performed to find therapeutic targets in the case of recurrent disease: about half of the centers test for gene fusions (60%) and tumor mutational burden (40%).

**Conclusion:**

Current notable interlaboratory variations as illustrated in this study should be reduced in order to refine diagnostics and improve precision oncology. In-house developed tests, standardized panels and routine application of broad gene panels all have their own advantages and disadvantages. Future research would be of interest to study the clinical impact of variation in diagnostic approaches.

## Introduction

The final diagnosis of a high-grade adult-type diffuse glioma is increasingly based on molecular characteristics of the tumor [1]. This dependence on molecular alterations has increased with the release of the fifth edition of the World Health Organization (WHO) classification of tumors of the central nervous system (WHO CNS5) in 2021 [2], which is largely based on the evidence provided by the Consortium to Inform Molecular and Practical Approaches to CNS Tumor Taxonomy– Not Officially WHO (cIMPACT-NOW) [3–5]. Consequently, the European Association of Neuro-Oncology (EANO) updated its guidelines for the clinical management of adult patients with diffuse gliomas and provided extensive recommendations on diagnosis and treatment, based on immunohistochemistry and additional molecular testing [6, 7]. Molecular characteristics can now overrule the diagnosis based on morphological characteristics, clearly illustrated in isocitrate dehydrogenase 1 and 2 (*IDH1/2*) and H3-wildtype diffuse gliomas where in adult patients regardless of the histology the presence of a *TERT*-promotor mutation, *EGFR*-amplification and/or a gain of chromosome 7 together with a loss of chromosome 10 (so called “+7/-10”), warrants the diagnosis of a glioblastoma, IDH wild-type (CNS WHO grade 4) [1, 3, 8–10]. This clinical value of molecular characteristics is also demonstrated in IDH-mutant astrocytomas in which the general favorability of low grade histology is overruled by the presence of homozygous *CDKN2A/B* deletion resulting in a grade 4 diagnosis [4, 11].

Besides methylome profiling, next generation sequencing (NGS) of tumor DNA is used in clinical practice to determine the molecular characteristics of a malignant brain tumor. Depending on the exact setup and protocol, NGS allows testing for mutations, gene fusions (especially RNA-based), copy number aberrations (CNAs) including loss of heterozygosity (LOH), and small insertions/deletions (InDel) [12–14]. To keep the costs of the NGS-tests reasonable, most molecular pathology laboratories currently apply targeted panels focusing on genes of interest for glioma diagnostics. These panels were generated and updated over time to keep up with the ever-evolving scientific literature and recommendations. Moreover, laboratories may use either lab developed tests (LDTs), i.e., custom-made panels, or commercial, standardized panels (also known as in vitro diagnostics (IVDs)). Hence, panels may vary significantly between centers, even when located within the same region. The interpretation of the molecular alterations occurs by the use of general oncogenetic concepts and the multiple databases. Although the workflows for these diagnostics are similar in different centers, reported genes and outcomes are not necessarily identical. The variation in NGS panel platforms as well as interpretation workflows can be expected to contribute to interlaboratory variation in the diagnostic work-up of and perhaps even in the treatment of adult patients with a malignant brain tumor.

This study aimed to evaluate the current practice in the application of NGS for patients with a high-grade adult-type diffuse glioma (HGG), both primary and recurrent, in the Netherlands in order to make recommendations on the clinical practice of genome-based diagnostics in patients with an HGG.

## Materials and methods

### Data collection

In the Netherlands, patients with a (suspected) brain tumor are referred to centers with neuro-oncological expertise. There are 14 neurosurgical centers that treat patients with glioblastoma, a diagnosis that is made approximately 1000 times a year in the Netherlands [15]. The diagnosis is definitive after the histological and molecular (‘histomolecular’) assessment by a (neuro)pathologist. Most often NGS is performed locally, but sometimes it is centralized, resulting in discrepancy between total number of neuro-oncological centers and specialized pathology departments involved in this study. The molecular reports are integrated in the morphology reports by the (neuro)pathologist, who is responsible for making accurate, ‘histomolecular’ diagnoses. Clinical scientists in molecular pathology (CSMPs) are responsible for the proper execution and interpretation of molecular assays. Together with (neuro)pathologists and clinical oncologists, CSMPs are important stakeholders in the molecular tumor board (MTB) in which rare and/or complex molecular information is being discussed and taken into account in a treatment advice for each patient [16, 17].

From April 2022 until July 2022, questionnaires were sent to (neuro)pathologists and CSMPs and qualitative data was collected on current NGS panel practice in the Netherlands. All (neuro)pathologists and CSMPs with experience with NGS were eligible for participation. Participants were selected from twelve centers in the Netherlands providing neuro-oncological pathology services, including seven academic centers, four peripheral hospitals and one independent pathology laboratory. One (neuro)pathologist and one CSMP (if any) were selected per center. Respondents were assured that answers on the questionnaires would be kept confidential and that the answers would be processed anonymously.

The questionnaire was designed in two different versions: one was sent to CSMPs to assess technical details on NGS panels, the other was sent to (neuro)pathologists to assess clinical aspects related to the ordering and reporting of NGS results. Part one of the questionnaire was about the practice for HGG at initial diagnosis, the second part was about the practice for HGG at recurrence and the final part evaluated future perspective regarding genome-based diagnostics. Questionnaires were sent via e-mail, and reminders were sent by e-mail or given by phone call up to two times to potential participants if they had not yet responded.

### Outcomes

The primary outcome was NGS panel practice for HGG at initial diagnosis, e.g. genes included in the different centers for diagnostic practice. Secondary outcomes were NGS panel practice for recurrent HGG (including the role of the MTB), and future perspectives (including expectations on future replacement of NGS by whole genome sequencing (WGS)).

### Statistical analysis

Categorical variables were reported using percentages and counts with the intention to qualitatively analyze the results. Calculations were based on total number of respondents for the specific questions; missing answers were taken out from the analyses. Therefore, total counts might vary per outcome. Figures were created using the open software environment *R*, version 4.2.1.

## Results

### Questionnaire response

Of the twelve centers, nine of them had their own CSMP services. The questionnaire was filled out by eleven (11/12, 92%) (neuro)pathologists and seven (7/9, 78%) CSMPs. In total, 78% (14/18) of the respondents answered all questions of the questionnaire, the remainder skipped only one or two questions. See Table 1 for a summary of the most important results.

**Table 1:** Summary of the most important results from the questionnaire

*Primary lesions*	
Neuro-oncology NGS panels per week per center, no. (%)^a^	
0–5	6 (86%)
5–10	1 (14%)
Panel origination, no. (%)	
Lab developed test	6 (86%)
Commercial test	1 (14%)
Latest panel update (%)	
Before 2019	1 (14%)
2019 or later	5 (71%)
Unknown	1 (14%)
CNAs analysed, no. (%)	
Yes	6 (86%)
No	1 (14%)
NGS applied by default, no. (%)	
Yes	4 (36%)
No	2 (18%)
Only in specific cases	5 (46%)
Markers always reported, no. (%)	
Diagnostic markers	11 (100%)
Prognostic markers	8 (73%)
Predictive markers	3 (27%)
Actionability	0 (0%)
*Recurrent lesions*	
Neuro-oncology NGS panels per week per center, no. (%)	
0–5	7 (100%)
5–10	0 (0%)
Composition molecular tumor board, no. (%)	
Clinical scientist in molecular pathology	6 (100%)
(Neuro)pathologist	2 (33%)
Neurologist	3 (50%)
Neurosurgeon	1 (17%)
Medical oncologist	5 (83%)
Other (e.g. clinical geneticist)	4 (67%)
CNAs analysed, no. (%)	
Yes	5 (83%)
No	1 (17%)
Goal(s) molecular diagnostics, no. (%)	
Diagnostic markers	1 (10%)
Therapeutic targets	8 (80%)
Gene fusions	6 (60%)
Tumor mutational burden	4 (40%)
Methylome profiling	1 (10%)
Other (e.g. microsatellite instability)	3 (30%)

^a^Total counts vary because the total number of respondents differed per question. CNAs: copy number aberrations. NGS: next generation sequencing

### Initial tumor

In the diagnostic process of an HGG, in 4/11 (36%) centers NGS is always applied by default, and in another 5/11 (46%) it is only used for specific patient groups, for instance patients aged under 55 or 60 years, when immunohistochemistry is not sufficient for the diagnosis of an IDH1 R132H wild-type glioblastoma. In 2/11 (18%) of the centers, NGS is not used by default, but rather methylome profiling for instance. When NGS is applied, most centers (9/11, 82%) always explicitly reported diagnostic markers (e.g., *IDH1/2*, *ATRX*, *TERT*), regardless the mutational status of the marker (e.g., ‘No mutation in *IDH1/IDH2* found’). Likewise, prognostic markers (e.g. *CDKN2A/B*) were always reported in 8/11 (73%) of the laboratories, in contrast to (not exclusively) predictive markers (e.g. *BRAF*, *EGFR*) (3/11, 27%) and details on actionability (0%).

**Fig. 1:**
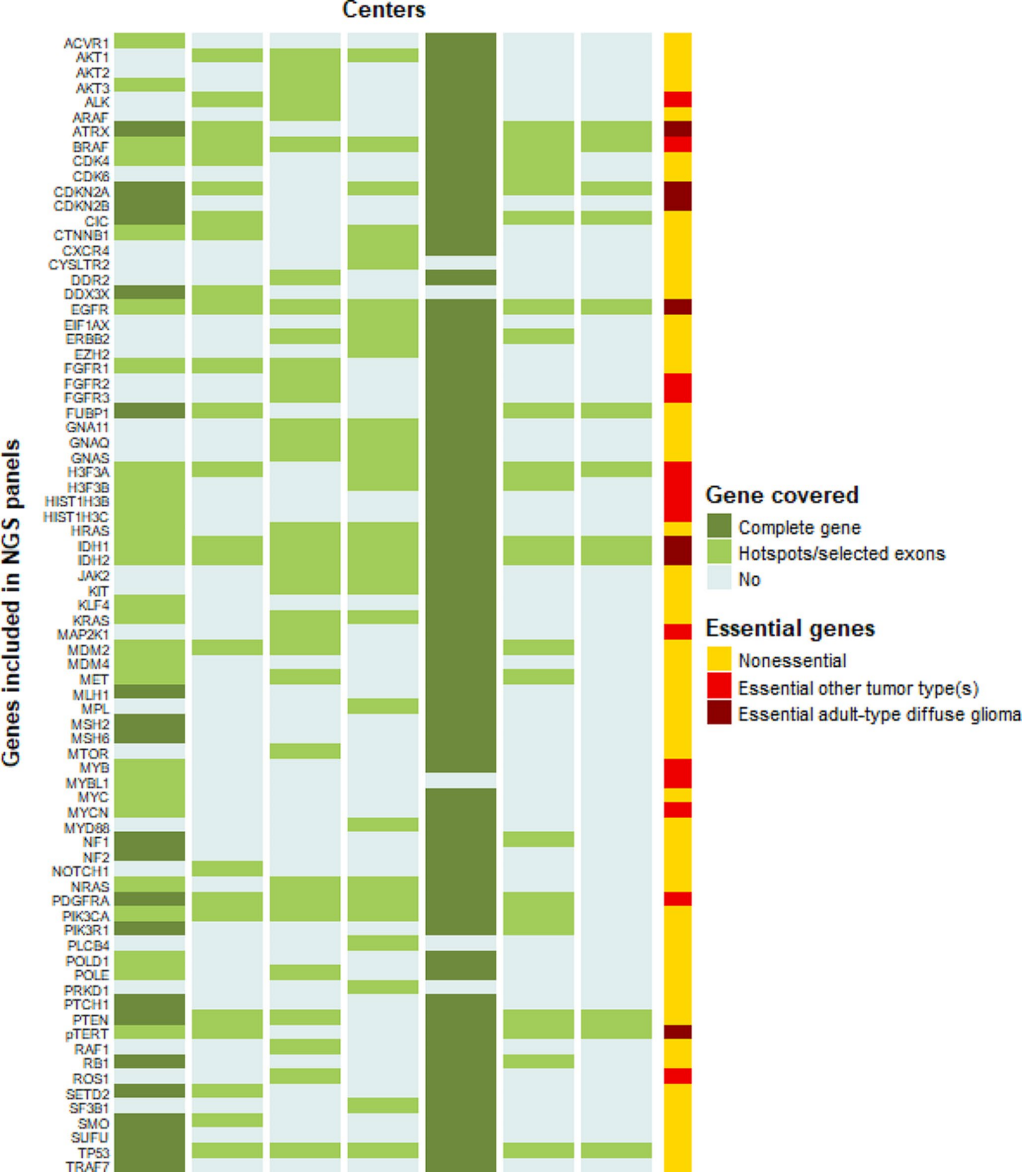
Heatmap overview of next generation sequencing (NGS) gene panels in different centers. Seven centers provided detailed panel information. For the center with the broad 500 gene panel by default, only those genes present in at least one of the other panels are depicted. Essentiality is based on the fifth edition of the World Health Organization classification of tumors of the central nervous system (WHO CNS5) [2, 18]

**Fig. 2:**
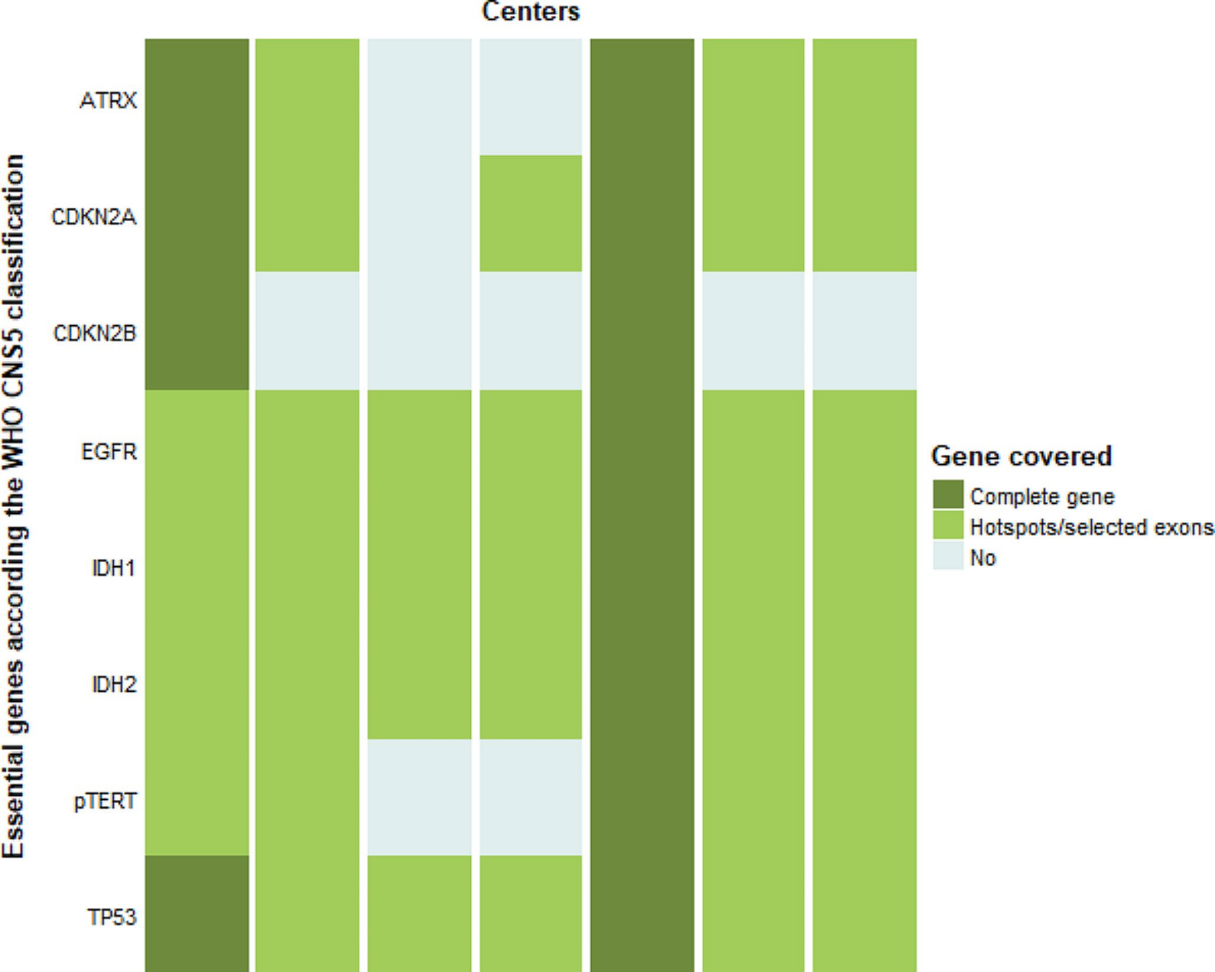
Heatmap overview of next generation sequencing (NGS) platforms in different centers regarding genes listed as essential for adult-type diffuse glioma diagnosis [2, 18]. WHO CNS5: fifth edition of the World Health Organization classification of tumors of the central nervous system

All but one (10/11, 91%) center used LDTs by default for the diagnosis of an HGG. The composition of the NGS gene panels for diagnosis of the initial tumor was different in each center (Fig. 1, panel composition obtained from the seven CSMPs), and numbers of genes included in the different panels ranged from 12 to 49 for the LDTs. One of the centers used a broad gene panel (TruSight Oncology 500, TSO500) containing 523 genes in the diagnostic setting; other centers would be able to do this by indication. No correlation was observed between the size of a center (based on national quality registries) and size of a panel. Regarding the genes essential for the diagnoses of adult-type diffuse gliomas according the WHO CNS5 classification [18], 2/7 (29%) covered all these eight genes (Fig. 2). Of the most relevant of these genes, *IDH1/2, TP53* and *EGFR* are covered by all panels whereas two panels did not cover mutations in the *TERT-*promotor. However, these centers test for *TERT*-promotor mutation via a separate test such as droplet digital polymerase chain reaction (ddPCR), whether or not at the request of the (neuro)pathologist. Only the broad gene panel covered complete genes, the LDTs were limited to hotspots.

### Recurrent tumor

In the case of molecular diagnostics for recurrent HGGs, 8/10 (80%, one respondent missing) of the centers apply genome sequencing to identify potential therapeutic targets. All centers have access to an MTB (whether it be in or outside their own infrastructure), but none of them discuss every patient after analysis of potential therapeutic targets. Selection is based on the molecular findings, for instance to discuss targeted treatment options, and discussion in MTBs is almost exclusively at the request of the treating physician. The composition of the MTB differs in each center, but always CSMPs and medical oncologists are members of the MTB [17].

Regarding the testing for potential therapeutic targets in recurrent lesions, the decision to apply these molecular diagnostics is a multidisciplinary decision, for instance made during regular multidisciplinary discussion attended by clinicians and (neuro)pathologists. Reasons for the use of additional molecular analysis in the case of a recurrent HGG include the absence of NGS in the primary setting, ambiguity in previous test results, the introduction of new molecular markers since the primary diagnosis, or a relatively young patient in a good condition (Karnofsky Performance Status (KPS) ≥ 70).

### Future perspectives

The majority (5/7, 71%) of the CSMPs expect updates of the current NGS panel within two years, with both diagnostic and therapeutic targets in small (17%) or broad (83%) NGS panels. 7/11 (64%) of the (neuro)pathologists do not expect a replacement of NGS by WGS for the diagnostics of adult HGG within five years, while 3/11 (27%) do expect this, and 1/11 (9%) do not know. Most important arguments for this skepticism towards WGS include the cost-effectiveness (7/8, 88%) and too much/irrelevant data to analyze (6/8, 75%). However, maximizing treatment options by WGS based diagnostics was an important argument for three (neuro)pathologists to see future importance of WGS within five years.

## Discussion

This study investigated the current practice in the application of NGS for patients with a high-grade adult-type diffuse glioma in the Netherlands. Of the seven centers that shared their information via the CSMPs, NGS panels were different in each center, with a wide range in the number of genes per panel.

In a country where molecular testing is relatively widely reimbursed, financial incentives are not likely to play an important role in the interlaboratory variation as found in our study. Explanatory factors could be, for example, local protocols or variable interest in experimental, molecularly targeted, therapeutic options. The variability in the composition of the panels as found in our study can also be explained by the finding that six of the seven panels were LDTs. These in-house produced tests result by definition in practice variation between different centers and frequently updating LDTs is difficult. A Dutch interview-based research investigated the application of diagnostics in hospital practice and found no straightforward explanation for the use of either LDTs or commercial tests [19]. However, that study showed that explanatory features of LDTs include the lower costs and the tailoring to the specific laboratory practices, compared to commercial panels. Importantly, commercial tests are not by definition superior to LDTs since commercial tests could not easily or quickly be updated (i.e., adapted to the newest molecular criteria), and they do not rule out the possibility of practice variation when it comes to the interpretation of test results.

Practice variation in the application of NGS for patients with HGG could possibly result in diagnostic variability and delayed diagnosis. Even though different centers most often end up with the same molecular information for the primary diagnosis after sequential, layered testing, this would be time and eventually cost consuming. Differences are more pronounced when tests are performed in order to find therapeutic targets in the case of recurrent disease. For example, about half of the centers test for gene fusions (60%) and tumor mutational burden (40%). Although the occurrence of targetable gene fusions in glioblastoma is low and treatment effectiveness in the context of expediency is still being investigated, patient selection for potential trial participation is reduced when testing is omitted [20, 21].

The variable, layered diagnostic process could potentially be solved by routine application of broad gene panels, supplemented by broad gene fusion tests for instance in the case of recurrent disease. Considerable advantages of generic, broad gene panels over LTDs include less need for updates, significantly less risks of omitting to test certain biomarkers, and time-efficiency. These advantages must be weighed against higher costs, potential difficulties with reimbursement, increased risk of unsolicited findings and the fact that broad gene panels are sometimes inferior in detecting CNAs (and especially deletions like *CDKN2A*).

In May 2022, the new In Vitro Diagnostic Medical Devices Regulation (IVDR) came into effect in the European Union with the goal to improve patient safety and to ensure that innovative medical devices remain available [22]. This IVDR, the implementation of which will gradually unfold, will also affect in-house produced tests leading to more standardization of the diagnostic practice. Although more strictly regulated, IVDR requirements should not impede the application of LDTs [23]. However, professionals express their worries about the impact of the IVDR possibly resulting in decreased innovativeness and increased costs and administrative work [19].

This study has some limitations to be mentioned. First, this online survey is a reflection of the current laboratory practice, of both initial and recurrent HGG, and standard protocols per center, and left little room for discussion. For instance, a center with a smaller diagnostic panel might deploy broader diagnostics by indication. Second, local approaches possibly will slightly differ between (neuro)pathologists and/or CSMPs, but our study did not require more than one (neuro)pathologist and one CSMP per center to test for this inter- and intraspecialty variation. Another limitation is that the current study design did not account for the multidisciplinary setting in which decisions on the treatment of brain tumor patients are made in Dutch practice. Finally, this study did not assess the impact on clinical practice after NGS analysis in the different centers.

To conclude, our study illustrates the current interlaboratory variation in the application of NGS panels for patients with a high-grade adult-type diffuse glioma, both at first diagnosis and in the recurrent setting. Reducing this practice variation by applying broad gene panels as a standard has the dual potential of refining the diagnostics and improving precision oncology. Future research would be of interest to study the clinical impact of variation in diagnostic approaches.

## Data Availability

No datasets were generated or analysed during the current study.
